# Cross-cultural adaptation of a questionnaire assessing the child and parents’ expectations of orthodontic treatment in Brazil

**DOI:** 10.1590/2177-6709.27.2.e222083.oar

**Published:** 2022-06-10

**Authors:** Mariana Gonzaga Erthal RIBEIRO, Emanuelle de Fátima Ferreira OLIVEIRA, Mariele Cristina Garcia PANTUZO, Maria Ilma de Souza Gruppioni CÔRTES, Ildeu ANDRADE

**Affiliations:** 1 Pontifícia Universidade Católica de Minas Gerais, Faculdade de Odontologia, Programa de Pós-Graduação em Odontologia (Belo Horizonte/MG, Brazil).; 2 Medical University of South Carolina, College of Dental Medicine, Department of Orthodontics (Charleston/SC, USA).

**Keywords:** Life expectancy, Orthodontics, Validation studies, Surveys and questionnaire

## Abstract

**Introduction:**

The Sayers and Newton questionnaire was developed in England to assess the child’s and parent’s expectations about orthodontic treatment.

**Objective:**

The aim of this study was to carry out the cross-cultural adaptation of the questionnaire for the Brazilian Portuguese language, to test its reliability, and to compare patients’ and their parents’ expectations of orthodontic treatment.

**Methods:**

After translation and cross-cultural adaptation, the questionnaire was applied to 98 patients (12-14 years), who had been referred for treatment, and their caregivers. The internal consistency of the instrument was assessed by Cronbach’s Alpha Coefficient and the test-retest reliability, by Intraclass Correlation Coefficient (ICC).

**Results:**

Internal reliability was confirmed by a Cronbach’s alpha coefficient of 0.75. Test-retest reliability revealed satisfactory reproducibility (ICC = 0.85). The results showed some significant differences between the expectations of the patients and their caregivers (*p* < 0.05). There were no significant gender differences (*p* > 0.05).

**Conclusions:**

The process of cross-cultural adaptation of the Sayers and Newton questionnaire for the Brazilian Portuguese was concluded. This study demonstrated that this instrument is reliable and applicable to assess the child’s and parent’s expectations about orthodontic treatment in Brazil, and it has acceptable psychometric properties.

## RESUMO

**Introdução:**

O questionário de Sayers e Newton foi desenvolvido na Inglaterra para avaliar as expectativas dos pacientes e de seus responsáveis quanto ao tratamento ortodôntico.

**Objetivo:**

O objetivo do presente estudo foi realizar a adaptação transcultural do questionário para a língua portuguesa do Brasil, testar sua confiabilidade e comparar as expectativas de pacientes e responsáveis em relação ao tratamento ortodôntico.

**Métodos:**

Após tradução e adaptação transcultural, o questionário foi aplicado a 98 pacientes (12-14 anos), encaminhados ao departamento de Ortodontia da PUC-MINAS para tratamento e de seus responsáveis. A consistência interna do instrumento foi avaliada pelo coeficiente alfa de Cronbach, e a confiabilidade teste-reteste, pelo coeficiente de correlação intraclasse (ICC).

**Resultados:**

A confiabilidade interna foi confirmada pelo coeficiente alfa de Cronbach de 0,75. A confiabilidade teste-reteste revelou reprodutibilidade satisfatória (ICC = 0,85). Os resultados mostraram algumas diferenças significativas entre as expectativas dos pacientes e de seus responsáveis (*p* < 0,05). Não houve diferenças significativas quanto ao sexo (*p* > 0,05).

**Conclusões:**

Foi concluído o processo de adaptação transcultural do questionário de Sayers e Newton para o português brasileiro. Esse estudo demonstrou que esse instrumento é confiável e aplicável para avaliar as expectativas dos pacientes e dos pais sobre o tratamento ortodôntico no Brasil e possui propriedades psicométricas aceitáveis.

## INTRODUCTION

A full understanding of patient motivation and expectations regarding orthodontic treatment leads to improved treatment plan design, better cooperation throughout therapy, and successful results.^1^ ^,^ ^2^ Expectations can be influenced by several factors, such as the differences between health systems, ethnic diversity, and cultural differences.^3^ ^-^ ^8^ As expectations are an important psychological factor, they can influence the evaluation of the patient regarding the quality of the treatment or the final satisfaction with the results.^9^ This is even more relevant in long-term treatments, for which esthetics is a significant component of treatment outcome. In addition, patients can be presumed to perceive the efficacy of treatment by comparing their expectations with their actual experiences.^5^

Previous studies have revealed that the expectations of patients and their parents in relation to orthodontic treatment are based on improving facial or dental appearance and improving health and oral function.^3^ ^,^ ^10^ In addition, improved attractiveness and psychological confidence are also benefits sought with therapy.^5^ However, they also have expectations regarding the events that will occur during this time, such as the duration of treatment, possible extractions, pain and discomfort with feeding, talking, and cleaning teeth. Some studies were performed after the initial consultation or during treatment^1^ ^,^ ^11^ and reported a belief that orthodontic treatment would enhance the appearance, self-confidence and oral function. Other studies that evaluated only the caregivers’ expectations^10^ reported an improvement of children’s self-esteem with treatment. Previous studies, however, have indicated that parental expectations differ and often outweigh those of patients, as parents expect greater improvements in their children than the children themselves.^7^ ^,^ ^12^ ^-^ ^14^ However, few studies have measured the expectations of patients and their caregivers before the initial appointment.^3^ ^,^ ^6^ ^,^ ^11^ In England, Sayers and Newton assessed the expectations of patients and their caregivers regarding orthodontic treatment prior to their initial consultation using a validated questionnaire.^6^ ^,^ ^12^ Their results showed similar expectations regarding orthodontic treatment.

The Sayers and Newton questionnaire (SNQ) was the first to provide a reliable measure of patients’ expectations prior the orthodontic treatment, and has been widely used in the literature.^6^ ^-^ ^16^ It was formulated according to cultural and ideological aspects of the English population, which hinders its application in the Brazilian population. As valid instruments in Portuguese are not yet available to evaluate the patients’ expectations before orthodontic treatment, it is necessary to translate and validate this questionnaire into this language.^12^

Translation and cultural adaptation of instruments are internationally recognized methods. The translation consists of obtaining a version in the chosen language that is semantically equivalent to the original language version. Cross-cultural adaptation is necessary when the instrument will be used in a population that is culturally different from the population used in the development of the original instrument. This process is important, since each society has characteristics that reflect its culture and differentiate it from others. Thus, it is necessary to propose the application of a data collection instrument with simple, clear language and equivalence in relation to cultural concepts.^12^

The development of a valid and reliable method of evaluating the expectation of orthodontic treatment will be useful in planning and implementing a more effective treatment. Moreover, it will help in the creation of a public policy that can implement Orthodontics in the Brazilian Health System. Orthodontists will be able to use the questionnaire to assess patients’ expectations and, possibly, determine a more appropriate treatment plan for the patient, which can avoid premature abandonment of treatment and dissatisfaction with the results.^4^ ^,^ ^13^ ^,^ ^15^

It was hypothesized that the SNQ, whose design was largely based on focus group interviews, was reliable and valid in the Brazilian Portuguese language. Thus, the objective of this study was to cross-culturally adapt the SNQ, to test the reliability and validity of the questionnaire in the Brazilian Portuguese language, and to compare patients’ and their parents’ expectations of orthodontic treatment.

## MATERIAL AND METHODS

### STUDY DESIGN AND ETHICAL CONSIDERATIONS

The methodology emphasizes the cross-cultural adaptation of the SNQ and its psychometric testing for test-retest reliability, internal consistency and construct validity. The study was approved by the Research Ethics Committee of the *Pontifícia Universidade Católica de Minas Gerais* (PUC MINAS, Brazil). All the participants signed the consent term. The present questionnaire validation process followed the steps suggested by Guillemin et al.^17^

### DESCRIPTION OF THE SAYERS AND NEWTON QUESTIONNAIRE

The SNQ consists of 10 questions that encompass the following domains: (1) content of the initial consultation, (2) type of device to be used, (3) problems expected during treatment, such as pain and difficulty in eating, (4) other people’s reactions to treatment, (5) total duration of treatment, (6) frequency of appointments, and (7) expectations with treatment outcome. Eight out of these questions should be answered using a 10-point scale ranging from “not at all likely” to “very likely,” where one corresponds to a very low expectation of a given event and ten signifies that the patient assesses that there are many chances of a certain event happening during his/her orthodontic treatment. The questions 8 and 9, which are related to treatment time and appointment frequency, respectively, should be answered according to a time scale.

### TRANSLATION AND CULTURAL ADAPTATION OF THE SAYERS AND NEWTON QUESTIONNAIRE

**Figure 1:**
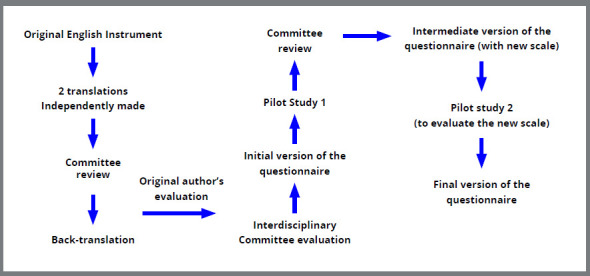
Flowchart with the steps of cross-cultural adaptation process.

The stages of this process are presented in Figure 1. Based on standard recommendations, the translation (English to Brazilian Portuguese) of the original questionnaire was done individually by two bilingual translators (both translators were native Brazilian Portuguese speakers, fluent in English and experts in epidemiological studies). In order to determine the concept and equivalence of both versions, they were evaluated by an expert committee of three Brazilian-born orthodontic professors, and three orthodontic residents of the Department of Orthodontics of the aforementioned institution (PUC MINAS), all of them fluent in Brazilian Portuguese and English, and with experience in dental research. This version was compared to the original version of the questionnaire, paying attention to the meaning of the words in the different languages ​​and identifying possible difficulties in understanding the questionnaire. Some questions were re-evaluated and rewritten, and through consensus, the initial version of the questionnaire was obtained. 

The back-translation was carried out separately by two native English orthodontic professors, who were blinded to the original instrument and were not informed about the purpose of the research, in order to allow them to realize the back-translation free of bias for the study. The back-translated version was emailed to the authors of the original instrument, who provided some minor suggestions. The back-translators and the expert committee approved their suggestions, and through consensus, the initial version of the questionnaire was obtained.

This initial version was then pre-tested in a pilot study with 12 randomly selected adolescents of both genders (aged 12-14 years) from the Department of Orthodontics at PUC MINAS. The questionnaire was applied through an individual interview during which the participants were asked about the comprehension of each question. The questions that presented an incomprehension index greater or equal to 20% of the total sample population were re-evaluated and reformulated in order to clarify the content of the questionnaire.

It was also necessary to modify the scale design used for the questionnaire responses. The visual analogue scale used in the original questionnaire did not present easy comprehension by the patients, who did not understand the degree of intensity inserted in the scale, nor did they know how to mark their responses on the scale. Therefore, a numerical 10-point Likert scale was chosen after being applied in a pilot test. It varied between “not at all likely” (1) to “extremely likely” (10).

This intermediate version of the questionnaire was again tested with 22 patients with the same characteristics of the first pilot study. After this second pilot study, where the index of understanding the questions reached 100%, the final Portuguese version of the questionnaire was then obtained.

### EVALUATION OF THE RELIABILITY OF THE PORTUGUESE VERSION OF THE SAYERS AND NEWTON QUESTIONNAIRE

To evaluate the reliability of the instrument, the final version of the questionnaire was applied to a convenience sample of 196 participants, 98 patients and 98 of their parents, who were awaiting orthodontic treatment at the Department of Orthodontics of PUC MINAS. Inclusion criteria were as follows: Patients aged between 12-14 years; no previous history of orthodontic treatment; subjects who were intellectually and physically capable of responding the instrument. The questionnaires were self-administered, but interviews were carried out prior to its application in an informal way by the first author in a non-clinical setting, with no time pressures, in order to explain patients and caregivers how to answer the SNQ. The children were interviewed separately from their parents. Each interview took approximately 10-15 minutes to complete. 

After verification of the data, the statistical analysis was performed. The reliability of the instrument was assessed using Cronbach’s alpha, with a value greater than 0.7 being acceptable. Test-retest reliability was assessed by calculating the intraclass correlation coefficient (ICC) with a two-way random effects model for the questionnaire score using the data from 20 children who were interviewed a second time by the same investigator 3 weeks following the first interview. The acceptable reliability of the instrument determines that all questions are necessary and cohesive for evaluation of the research data. Floor and ceiling effects were evaluated by determining the proportion of patients/parents who answered each one of the questions with the worst or the best expectation. 

All the results were considered significant for a probability of significance of less than 5% (*p* < 0.05). A paired *t*-test was used to compare the expectations regarding orthodontic treatment between patients and caregivers. Furthermore, the *t*-test was used to compare the two independent groups regarding the measure of a variable of interest of the scalar type.

## RESULTS

Characterization of the sample in relation to personal data is described in Table 1.

**Table 1:** Characterization of the sample in relation to personal data (Mean age = 12.8 years, SD = 0.8.

Variable	Frequency	
	n	%
Patient's gender		
Female	51	52.0
Male	47	48.0
Total	98	100.0
Patient's age		
12 years	41	41.9
13 years	36	36.7
14 years	21	21.4
Total	98	118.0
Parent’s age		
30 - 39 years	36	36.7
40 - 49 years	37	37.8
50 - 59 years	20	20.4
60 years or more	5	5.1
Total	98	118.0

### INTERNAL CONSISTENCY

The value of Cronbach’s alpha was greater than 0.75, showing that the questionnaire had an acceptable internal consistency (Table 2). The overall inter-item value was 0.76 and the corrected item-total correlation of > 0.3 was achieved in over 50% of the questions, producing a good level of internal consistency. Test-retest reliability revealed satisfactory reproducibility (ICC = 0.85).

**Table 2:** Internal consistency analysis of the final version of the questionnaire.

Questions	Evaluated group					
	Patients		Parents		Total sample	
	Item-total correlation	Alpha if deleted item	Item-total correlation	Alpha if deleted item	Item-total correlation	Alpha if deleted item
1a	0.290	0.765	0.397	0.745	0.260	0.776
1b	0.093	0.775	0.196	0.758	0.180	0.779
1c	0.019	0.779	0.455	0.744	0.260	0.775
1d	0.371	0.759	0.453	0.741	0.417	0.766
1e	0.459	0.753	0.364	0.748	0.413	0.766
1f	0.246	0.767	0.388	0.747	0.336	0.771
2a	0.213	0.769	0.263	0.755	0.248	0.776
2b	0.405	0.758	0.433	0.744	0.386	0.768
2c	0.407	0.757	0.288	0.754	0.367	0.769
2d	0.362	0.760	0.346	0.749	0.379	0.768
2e	0.385	0.760	0.111	0.762	0.248	0.775
3	0.298	0.764	0.015	0.769	0.159	0.780
4	0.180	0.771	0.046	0.770	0.108	0.784
5	-0.060	0.785	0.189	0.760	0.070	0.786
6	0.140	0.774	0.114	0.766	0.142	0.783
7	0.387	0.759	0.293	0.753	0.352	0.771
10a	0.198	0.770	0.308	0.756	0.250	0.777
10b	0.428	0.761	0.305	0.755	0.391	0.772
10c	0.429	0.755	0.477	0.740	0.494	0.760
10d	0.453	0.753	0.480	0.739	0.503	0.759
10e	0.495	0.750	0.342	0.749	0.457	0.763
10f	0.442	0.754	0.522	0.737	0.516	0.758
10g	0.586	0.743	0.328	0.750	0.497	0.760
Total	0.771		0.760		0.779	

### EXPECTATION OF PATIENTS AND CAREGIVERS

Patients and their caregivers presented some significant differences (*p* < 0.05) regarding the expectation of orthodontic treatment (Table 3).

**Table 3:** Comparative analysis between patients and parents regarding the expectation of orthodontic treatment.

Questions	Evaluated group		P	Effect Size
	Patients ( $\bar{x}$ ± sd)	Parents ( $\bar{x}$ ± sd)		
1a	58.5 ± 32.7	45.3 ± 36.0	0.003	0.38
1b	72.7 ± 26.1	80.9 ± 25.6	0.023	0.32
1c	74.3 ± 27.0	84.8 ± 23.9	0.005	0.41
1d	66.0 ± 29.9	70.8 ± 31.3	0.288	0.16
1e	55.7 ± 33.2	59.9 ± 33.4	0.366	0.13
1f	72.9 ± 27.4	79.9 ± 27.4	0.042	0.26
2a	74.5 ± 27.6	78.3 ± 28.1	0.353	0.14
2b	82.0 ± 24.9	79.4 ± 27.4	0.466	0.10
2c	35.1 ± 31.7	45.7 ± 35.9	0.015	0.31
2d	26.4 ± 26.6	36.6 ± 31.7	0.007	0.35
2e	21.1 ± 23.4	22.8 ± 23.0	0.575	0.07
3	21.8 ± 24.2	24.7 ± 26.5	0.441	0.11
4	49.8 ± 29.6	50.2 ± 31.3	0.913	0.01
5	47.8 ± 29.2	49.4 ± 31.2	0.773	0.05
6	43.8 ± 30.8	48.7 ± 32.6	0.245	0.15
7	78.8 ± 24.5	82.4 ± 20.9	0.241	0.16
10a	96.7 ± 10.4	97.2 ± 11.0	0.719	0.05
10b	92.6 ± 16.3	96.6 ± 13.8	0.025	0.27
10c	57.2 ± 34.5	78.9 ± 29.6	< 0.001	0.68
10d	52.6 ± 36.4	71.4 ± 32.4	< 0.001	0.55
10e	60.9 ± 34.4	75.2 ± 29.4	< 0.001	0.45
10f	61.7 ± 35.5	80.7 ± 29.5	< 0.001	0.58
10g	66.6 ± 33.6	81.3 ± 29.2	< 0.001	0.47

The probability of significance (p) refers to the Student *t*-test for paired samples.

The comparison between gender expectations revealed no significant differences between these two groups (Table 4). In addition, no significant differences were found between the caregivers from male and female patients (Table 5).

**Table 4:** Comparative analysis between female and male patients regarding the expectation of orthodontic treatment.

Questions	Patient’s Gender		P	Effect Size
	Female (x ± sd)	Male (x ± sd)		
1a	54.5 ± 31.7	62.8 ± 33.5	0.214	0.25
1b	71.6 ± 26.6	73.8 ± 25.8	0.671	0.09
1c	76.7 ± 27.4	71.7 ± 26.6	0.366	0.18
1d	62.0 ± 32.1	70.4 ± 26.9	0.159	0.28
1e	61.8 ± 32.9	49.1 ± 32.7	0.060	0.38
1f	77.5 ± 26.4	67.9 ± 28.0	0.085	0.35
2a	75.9 ± 28.2	73.0 ± 27.0	0.604	0.10
2b	80.4 ± 23.7	83.8 ± 26.3	0.500	0.14
2c	38.8 ± 34.5	31.1 ± 28.2	0.225	0.25
2d	28.6 ± 30.2	24.0 ± 22.2	0.392	0.17
2e	24.7 ± 26.4	17.2 ± 19.2	0.111	0.32
3	23.7 ± 25.0	19.8 ± 23.4	0.422	0.16
4	53.1 ± 28.8	46.2 ± 30.3	0.247	0.24
5	48.2 ± 30.0	47.4 ± 28.6	0.888	0.03
6	45.3 ± 31.5	42.1 ± 30.4	0.614	0.10
7	78.8 ± 24.9	78.7 ± 24.5	0.984	0.00
10a	97.8 ± 6.4	95.5 ± 13.5	0.289	0.22
10b	92.5 ± 16.5	92.6 ± 16.3	0.999	0.00
10c	60.0 ± 34.9	54.3 ± 34.1	0.412	0.17
10d	53.9 ± 37.2	51.1 ± 35.9	0.700	0.08
10e	60.8 ± 35.1	61.1 ± 34.1	0.968	0.01
10f	62.4 ± 35.4	61.1 ± 35.9	0.859	0.04
10g	67.1 ± 35.7	66.2 ± 31.5	0.896	0.03

The probability of significance (p) refers to Student’s t-test for independent samples.

**Table 5:** Comparative analysis between male and female patients’ parents regarding the expectation of orthodontic treatment.

Questions	Patient’s Gender		P	Effect Size
	Female (x ± sd)	Male (x ± sd)		
1a	48.6 ± 36.1	41.7 ± 36.0	0.344	0.19
1b	81.2 ± 27.0	80.6 ± 24.3	0.917	0.02
1c	83.9 ± 24.6	85.7 ± 23.4	0.708	0.08
1d	71.6 ± 29.4	70.0 ± 33.4	0.803	0.05
1e	58.4 ± 30.8	61.5 ± 36.2	0.655	0.09
1f	84.1 ± 23.6	75.3 ± 30.6	0.117	0.32
2a	74.3 ± 31.8	82.6 ± 23.0	0.143	0.30
2b	77.1 ± 28.1	81.9 ± 26.7	0.382	0.18
2c	45.9 ± 37.7	45.5 ± 34.3	0.962	0.01
2d	37.8 ± 33.1	35.3 ± 30.3	0.694	0.08
2e	22.9 ± 23.5	22.6 ± 22.6	0.934	0.02
3	24.3 ± 27.9	25.1 ± 25.2	0.883	0.03
4	47.8 ± 33.4	52.8 ± 28.9	0.437	0.16
5	51.6 ± 33.4	47.0 ± 28.9	0.472	0.15
6	46.1 ± 34.5	51.5 ± 30.4	0.412	0.17
7	81.8 ± 22.1	83.2 ± 19.7	0.736	0.07
10a	97.8 ± 12.7	96.6 ± 8.9	0.573	0.11
10b	97.5 ± 13.7	95.7 ± 13.9	0.543	0.12
10c	79.4 ± 30.2	78.3 ± 29.3	0.853	0.04
10d	70.0 ± 33.9	73.0 ± 30.9	0.650	0.09
10e	74.3 ± 29.4	76.2 ± 29.7	0.757	0.06
10f	77.5 ± 31.2	84.3 ± 27.4	0.253	0.23
10g	82.2 ± 29.4	80.4 ± 29.3	0.771	0.06

The probability of significance (p) refers to Student’s t-test for independent samples.

Most participants were not able to specify the total treatment time that they would need to wear the orthodontic devices. In addition, regarding the periodicity during which the patients should attend appointments, a diversity of perceptions was observed (Table 6).

**Table 6:** Characterization of patients and caregivers regarding the duration of treatment and the frequency of appointments.

Variable	Group	
	Patient	Parents
Duration of treatment		
4 years	15 (15.3%)	6 (6.1%)
3.5 years	4 (4.1%)	2 (2.0%)
3 years	12 (12.2%)	17 (17.3%)
2.5 years	7 (7.1%)	10 (10.2%)
2 years	13 (13.3%)	18 (18.4%)
1.5 yeras	6 (6.1%)	3 (3.1%)
1 year	11 (11.2%)	8 (8.2%)
6 months	4 (4.1%)	0 (0.0%)
3 months	1 (1.0%)	0 (0.0%)
Don’t know	25 (25.5%)	34 (34.7%)
Total	98 (100%)	98 (100%)
Frequency of appointments		
Every 8 months	2 (2.0%)	0 (0.0%)
Every 6 months	1 (1.0%)	1 (1.0%)
Every 3 months	7 (7.1%)	4 (4.1%)
Every 2 months	13 (13.3%)	11 (11.2%)
Every 6 weeks	8 (8.2%)	4 (4.1%)
Every 4 weeks	15 (15.3%)	36 (36.7%)
Every 2 weeks	12 (12.2%)	4 (4.1%)
Every week	8 (8.1%)	8 (8.2%)
Twice a week	4 (4.1%)	1 (1.0%)
Don't know	28 (28.6%)	29 (29.6%)
Total	98 (100.0%)	98 (100.0%)

### FLOOR AND CEILING EFFECTS

Table 7 shows the answer options that denotes the worst and the best expectation for each one of the questions on the research instrument. Since it has a total of 23 questions, the floor and ceiling effects were constructed by observing the frequency distribution of the total answers for worst and best expectations (Table 8). None of the respondents (patient and parent) exhibited extreme positive and extreme negative expectations regarding orthodontic treatment in all questions. Among the patients, 10.2% demonstrated an extremely positive expectation in 7 out of the 23 items evaluated, and 21.4% demonstrated an extremely negative expectation in 2 out of the 23 items assessed. Among the parents, 10.2% responded with an extremely negative expectation to 10 out of the 23 questions, and 18.4% of the parents answered with extremely positive expectation in 3 out of the 23 questions. 

**Table 7:** Definition of the answer option that denotes the worst and the best expectation for each one of the questions on the research instrument.

Question	Answer option		Question	Answer option	
	Worst expectation	Best expectation		Worst expectation	Best expectation
1a	10	1	4	10	1
1b	1	10	5	10	1
1c	1	10	6	10	1
1d	1	10	7	1	10
1e	1	10	10a	1	10
1f	10	1	10b	1	10
2a	1	10	10c	1	10
2b	1	10	10d	1	10
2c	1	10	10e	1	10
2d	1	10	10f	1	10
2e	10	1	10g	1	10
3	10	1			

**Table 8:** Frequency distribution of patients’ and parents’ number of questions answered as the worst and the best expectation.

Number of questions	Patients		Parents	
	Worst Expectation n (%)	Best expectation n (%)	Worst Expectation n(%)	Best expectation n (%)
0	1 (1.0)	14 (14.3)	2 (2.0)	18 (18.4)
1	2 (2.0)	10 (10.2)	0 (0.0)	13 (13.3)
2	9 (9.2%)	21 (21.4)	6 (6.1)	13 (13.3)
3	3 (3.1)	12 (12.2)	4 (4.1)	18 (18.4)
4	9 (9.2)	10 (10.2)	6 (6.1)	15 (15.3)
5	7 (7.1)	11 (11.2)	3 (3.1)	7 (7.1)
6	9 (9.2)	6 (6.1)	4 (4.1)	5 (5.1)
7	10 (10.2)	5 (5.1)	4 (4.1)	5 (5.1)
8	8 (8.2)	4 (4.1)	5 (5.1)	2 (2.0)
9	8 (8.2)	2 (2.0)	8 (8.2)	0 (0.0)
10	6 (6.1)	1 (1.0)	10 (10.2)	2 (2.0)
11	6 (6.1)	0 (0.0)	7 (7.1)	0 (0.0)
12	3 (3.1)	0 (0.0)	8 (8.2)	0 (0.0)
13	4 (4.1)	1 (1.0)	9 (9.2)	0 (0.0)
14	8 (8.2)	1 (1.0)	4 (4.1)	0 (0.0)
15	3 (3.1)	(0.0)	4 (4.1)	0 (0.0)
16	1 (1.0)	(0.0)	2 (2.0)	0 (0.0)
17	1 (1.0)	(0.0)	5 (5.1)	0 (0.0)
18	0 (0.0)	(0.0)	4 (4.1)	0 (0.0)
19	0 (0.0)	(0.0)	2 (2.0)	0 (0.0)
20	0 (0.0)	(0.0)	1 (1.0)	0 (0.0)
21	0 (0.0)	(0.0)	0 (0.0)	(0.0)
22	0 (0.0)	(0.0)	0 (0.0)	(0.0)
23	0 (0.0)	(0.0)	0 (0.0)	(0.0)
Total	98 (100.0)	98 (100.0)	98 (100.0)	98 (100.0)

## DISCUSSION

A reliable measure of orthodontic expectations for children presenting with unrealistic expectations is helpful in effective treatment planning, consent and quality of the orthodontic treatment provided. The lack of instruments such as this one in Brazil restrains researchers to two possibilities: (1) developing a new instrument or (2) translating, adapting and validating an existing one. The disadvantages of the first option are the high cost, prolonged research time and, the limitations in terms of comparisons with data from other countries. Therefore, the second alternative is considered more economic, efficient and practical.

Studies that evaluate the expectations of orthodontic treatment are scarce in the literature and have been developed in English-speaking countries.^6^ ^,^ ^7^ ^,^ ^8^ ^,^ ^11^ ^,^ ^16^ ^,^ ^18^ In order to be used in a non-English population, instruments need to be translated, adapted, and validated. This process should follow internationally accepted methods to ensure that the new language versions resulting from the original questionnaires can be used in international comparative studies.^19^

The SNQ has been widely used in other studies to evaluate patients’ expectations on orthodontic treatment.^6^ ^,^ ^7^ ^,^ ^8^ ^,^ ^11^ ^,^ ^15^ ^,^ ^18^ However, this was its first cross-cultural adaptation in another language. The process of translation and cultural adaptation of the questionnaire was carefully conducted following the criteria of Guilhemin et al.^17^ The results showed a translated version similar to the original, highlighting the adequacy of the Portuguese version of the instrument. Test-retest reliability indicated adequate internal reliability. Therefore, all questions in the Brazilian Portuguese questionnaire are considered essential for assessing patients’ expectancies in relation to orthodontic treatment, as well as possessing a logical sequence for the evaluation of the instrument.^20^

The SNQ uses a numerical Likert scale, in which the participants answered the questionnaire individually and separately and without any external interference. The need to apply the instrument individually demonstrates the autonomy of the individuals and how they express their opinions and perceptions. A previous study used qualitative telephone interviews to elaborate a questionnaire, which can be a questionable method.^10^ However, it would be interesting if new studies evaluated the effects of different forms of application, such as through interviews realized by the patient’s own orthodontist or dental assistant.

Previous studies have measured the subjects’ expectations about orthodontic treatment only after the initial appointment or during treatment, which introduces a bias for the results, since the patients after the initial consultation have some information about the treatment and, therefore, their expectations may be influenced.^6^ This study measured patients’ expectations before orthodontic appointment or treatment, because the patient responses were based only on previous information that he/she had regarding orthodontic treatment.

Reliability is defined as an assessment of the reproducibility and consistency of an instrument.^21^ The reliability of a study is threatened by bias and error.^22^ ^-^ ^24^ In this study, the bias could have been a result of personal characteristics, that is, the mood (people in low spirits may under-estimate their health status) and self-esteem of the participants at the time of application of the questionnaire may have influenced their expectations. Another possibility may have been the participant’s way of answering the questions, because they may not have cared about the answer or even tried to guess the correct answer, and with that, did not express their true expectations. In this study, bias could have also resulted from random measurement error (the respondent guesses the answer or gives an unpredictable response), recall (memory) bias (participants remembering responses from questionnaire), and response style bias (participants responding to questions in the same manner regardless of the question). In addition, there may have been a selection bias, since the concentration of participants at a given location using a convenience sample may also be considered a limitation of the study. Further studies should be performed to apply the questionnaire in a more representative sample and in multi-centers to confirm and compare the reliability of the instrument. 

Internal consistency was tested using Cronbach’s alpha in regards to the overall inter-item and item-total correlations. A previous study^10^ used Cronbach’s alpha to assess the reliability of their questionnaire. However, these authors measured parents’ expectations of orthodontic treatment, but not children’s expectations.

Similarly to other studies, the present results found significant differences (*p* < 0.05) between the patients’ and caregivers’ expectations.^7^ ^,^ ^11^ ^,^ ^13^ ^,^ ^14^ These findings refute previous studies that evaluated the patients’ expectations by addressing the questions that were asked to the parents, and assumed that patients had similar expectations to their caregivers.^22^ In addition, these findings confirm the need to evaluate people individually to determine individuals’ autonomy. The expectation of an orthodontic appliance installed at the initial appointment was significantly higher for the patients than for the patients. As found in the UK questionnaire application^6^ and in another study^7^, the expectations of the caregivers for the first visit were more realistic than those of the patients, who often expected to have orthodontic appliances installed on the same day. Regarding the need for the use of extra-oral devices and dental extractions during orthodontic treatment, this study showed that the caregivers’ expectation was significantly higher than their children’s, which is contrary to the results of the application of this questionnaire in the United Kingdom and other studies.^6^ ^,^ ^7^ ^,^ ^11^ ^,^ ^25^ They revealed that both patients and caregivers had low expectations concerning the need for extra-oral appliances or dental extractions. Therefore, as in other studies, this may explain the low cooperation with the use of extra-oral devices.

This study observed that the patients and their caregivers did not present significant differences (*p* > 0.05) when expectations of pain and chewing impairment associated with orthodontic treatment were evaluated, as both believed that there would not be many food restrictions, and that orthodontic treatment would, at some point, be painful. These results differ from the study by Sayers and Newton,^6^ as patients expected significantly greater restrictions in relation to the types of food and beverages they could consume during orthodontic treatment compared to their caregivers.^6^ Other studies^11^ ^,^ ^25^ have reported that participants underestimated the changes they need to make in their diet as a result of the pain associated with orthodontic treatment.

The present study points out that the expectations of the caregivers were greater than those of their children regarding the orthodontic treatment results, such as aligned teeth, a better smile, improved chewing and speech, and better career prospects of the patients. In addition, the caregivers expected a greater increase in social trust as a result of orthodontic treatment. These results corroborate with those found in previous studies^6^ ^,^ ^25^ ^-^ ^27^, which demonstrated that the need for orthodontic treatment is mainly determined by a concern with appearance, and the caregivers expected to improve the appearance of their children.^22^ ^-^ ^28^

Some studies have shown that the gender variable can influence people’s expectations. Women tend to have higher expectations regarding orthodontic treatment than men.^11^ ^,^ ^24^ ^,^ ^28^ However, the present results, which are in agreement with other studies,^7^ ^,^ ^28^ did not present significant differences (*p*> 0.05) between genders. The present study sample could not be divided according to ethnicity, as in the study of Sayers and Newton,^6^ since the Brazilian population presents a great miscegenation. Other limitations of this study may include the use of a convenience sample and the absence of construct validity.

According to Sayers and Newton,^6^ the caregivers appeared to be more informed about the duration of orthodontic treatment than the children. However, the present results demonstrated a lack of knowledge of what a real orthodontic treatment is, for both parts, which corroborates with the results of previous studies.^7^ ^,^ ^11^

After the cultural adaptation process, the Portuguese questionnaire will be capable of evaluating the expectations of patients and their caregivers regarding orthodontic treatment, which can be used to help the consent process and treatment planning. It is also important to emphasize the importance of evaluating patients individually and separately from their caregivers, but with special attention to the moment of application of the questionnaire, in order to correctly explain the purpose of the instrument, assuring that the participants are comfortable and aware of the importance of expressing their perceptions regarding orthodontic treatment. Moreover, it is worth mentioning that there is a need to develop an instrument that can evaluate the expectations of patients in all age groups on orthodontic treatment.

## CONCLUSIONS

In conclusion, significant differences were found between patients’ and caregivers’ expectations for orthodontic treatment. There were no significant differences between gender expectations, nor were there significant differences in the caregivers’ expectations of the male and female patients.

The internal consistency of the process of translation and cross-cultural adaptation of the Portuguese version of the SNQ was satisfactory. The Portuguese version of the questionnaire developed by Sayers and Newton can be used as an instrument to evaluate expectations regarding orthodontic treatment for Brazilian children from 12 to 14 years.
